# Supplementary material for: Overexpression of TCP5 or Its Dominant Repressor Form, TCP5-SRDX, Causes Male Infertility in Arabidopsis
Source: Int J Mol Sci. 2025 Feb 20;26(5):1813. doi: 10.3390/ijms26051813 (PMC11899387; doi:10.3390/ijms26051813)
Supplement: Supplementary file 1 [file ijms-26-01813-s001.zip › ijms-3368637-supplementary.pdf]

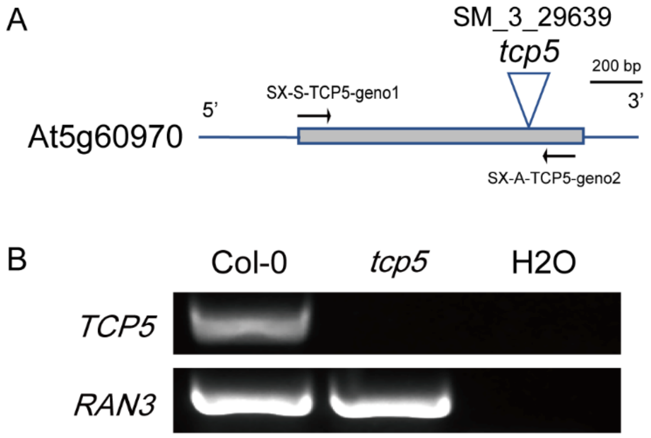

Figure S1. Confirmation of the *TCP5* T-DNA mutant. (A) *TCP5* gene structural diagram showing the position of the T-DNA insertion. (B) RT-PCR confirming that *tcp5* was a null mutant.

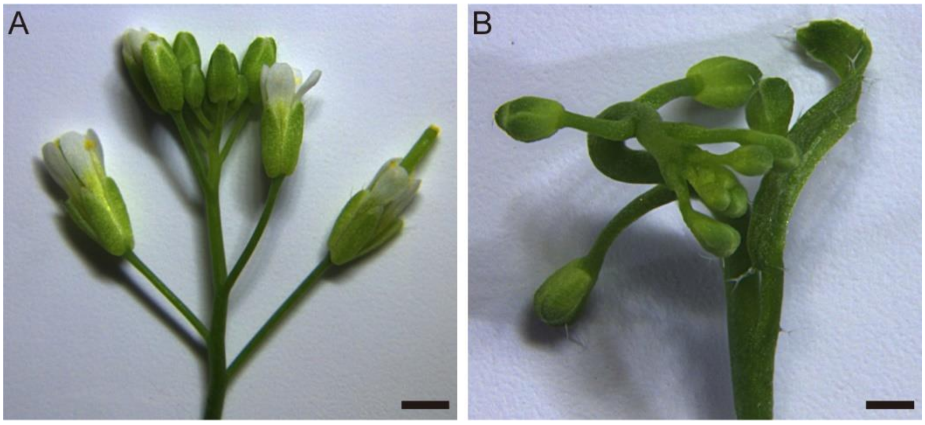

Figure S2. Apical structure of the inflorescence in *pUBQ10:TCP5* plant. (A) Wild-type inflorescence. (B) Inflorescence in *pUBQ10:TCP5* plant with twisted structure at the top. Scale bars: 1 mm (A, B)

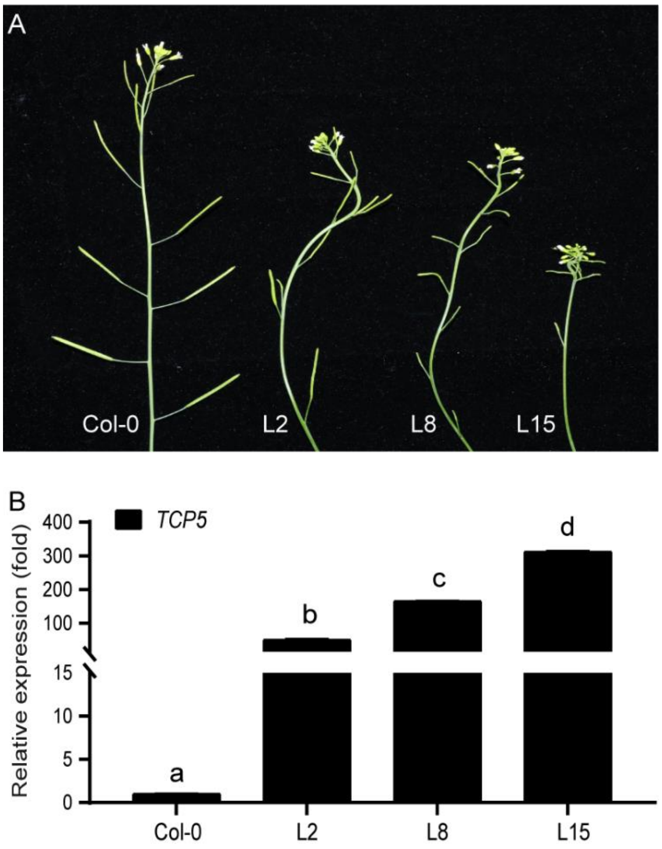

Figure S3. Correlation between *TCP5* expression level and sterility severity in *pUBQ10:TCP5* plants. (A) L2, L8, and L15 with distinct fertility strengths. (B) *TCP5* expression levels in L2, L8, and L15 lines compared to the wild-type. L15 exhibited the highest *TCP5* expression and the most severe sterile phenotype, indicating a positive correlation. Different letters represent significant differences in expression.

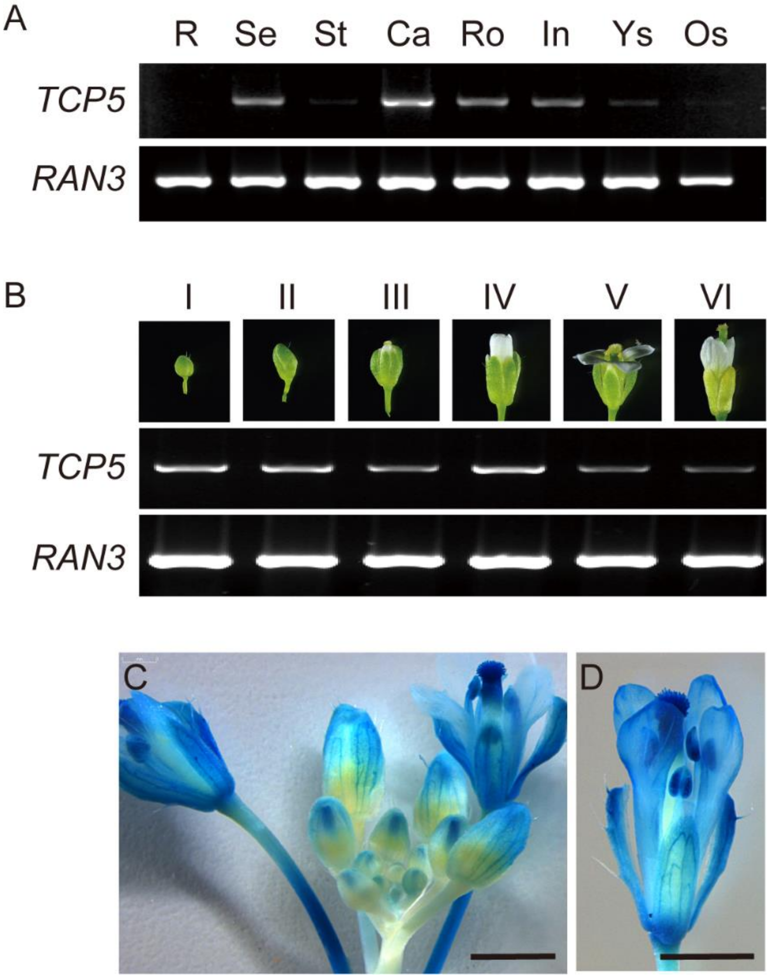

Figure S4. *TCP5* expression profile. (A) T-PCR of various organs. (B) RT-PCR of various flower buds and open flowers ［55］. (C, D) GUS-stained inflorescence and open flower of *pTCP5:GUS* plants.

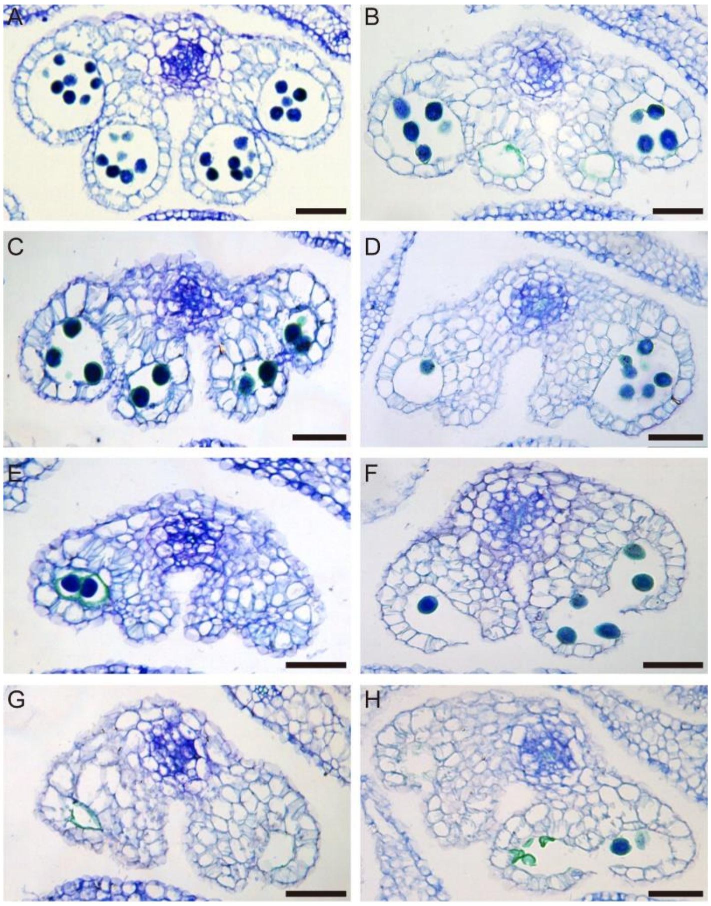

Figure S5. Anther cross-sections of *TCP5-SRDX* transgenic plants. (A) Cross-section of a wild-type anther at stage 11. (B-H) Cross-sections of *TCP5-SRDX* anthers. Anthers at stages 11 (B, D) and 13 (F, H) in *pSPL8:TCP5-SRDX* plants. Anthers at stage 12 (C, E, G) in *pUBQ10:TCP5-SRDX* plants. Scale bar: 50 μm.

Table S1. List of primers used in this study.

Primer name	Gene	Sequence（5'→3'）	Purpose
SM-line-Spm32		TACGAATAAGAGCGTCCATTTTAGAGTGA	Genotyping
SX-F-TCP5-geno1	*TCP5*	ATGAGATCAGGAGAATGTGATGAAG	
SX-R-TCP5-geno2		TCCGTATCATGATTCGAGCTCATC	
LT-F-TCP5-attB1	*TCP5*	GGGGACAAGTTTGTACAAAAAAGCAGGCTTAATGAGATCAGG AGAATGTGATGAAG	Entry vector
SX-R-TCP5-attB2-wo		GGGGACCACTTTGTACAAGAAAGCTGGGTGAGAATCTGATTC ATTATCGCTAC	
SX-F-pSPL8-StuI	*pSPL8*	GCaggcctTATGCATAATACTTGCGCAG	Promoter
SX-R-pSPL8-XhoI		TGTATctcgagTTGCAATCCCGAGGAAGATCTCTCTC	
SX-F-pTCP5-XbaI	*pTCP5*	AGtctagaTTAGTATTGCATATAGCCTATAC	
SX-R-pTCP5-SmaI		TGCAcccgggCTCTTTAATTCACTCAACAAG	
SX-F-TCP5-insitu1	*TCP5*	GCGGaattaaccctcactaaagggATGAGATCAGGAGAATGTGATG	RNA *in situ* hybridization
SX-R-TCP5-insitu1		GCTCgtaatacgactcactatagggcTCAAGAATCTGATTCATTATCGC	
LT-F-TCP5	*TCP5*	CAGCAAAGTATGTACAGTCCGTG	qRT-PCR
SX-R-TCP5		TCATCTTTTGCTGCTTCGAGTAAC	
LT-F-IRX1	*IRX1*	ACGGAGAGTTCTTTGTGGCT	
LT-R-IRX1		GGTCTGTGTTGGAACAATGG	
LT-F-IRX3	*IRX3*	CAGGCGTACTCACAAATGCT	
LT-R-IRX3		TGTCAATGCCATCAAACCTT	
LT-F-IRX5	*IRX5*	GGATCAGCTCCGATCAATTT	
LT-R-IRX5		ACCACAAAGGACAATGACGA	
SX-F-IRX8	*IRX8*	CATGGGCTTATGGAATGAATG	
SX-R-IRX8		AGCTGCCACAAACTCAGGTC	
SX-F-IRX9	*IRX9*	TACTTTGGGACCCTGAGAGATG	
SX-R-IRX9		ACAATCTTGTGCCGGAAGTC	
SX-F-CESA1	*CESA1*	GGGCAGTTAAGGTGATTCCA	
SX-R-CESA1		TTGGGTCCACATCTTCTTCC	
LT-F-MYB26	*MYB26*	CCATGGATGTTGGAGCTCTGTT	
LT-R-MYB26		GCTTCCACGTTTAAGATCAGGTCT	
LT-F-PAL4	*PAL4*	GCCGCCGCAATTATGGAACAT	
LT-R-PAL4		GCTGCTCTGCGCTTTGGACA	
SX-F-C3H	*C3H*	GTTGCTAACGTTGAAGGATCAG	
SX-R-C3H		TTCCGCTGTTATCGCTGTC	
LT-F-C4H	*C4H*	ACTGGCTTCAAGTCGGAGAT	
LT-R-C4H		ACACGACGTTTCTCGTTCTG	
LT-F-4CL1	*4CL1*	TCAACCCGGTGAGATTTGTA	
LT-R-4CL1		TCGTCATCGATCAATCCAAT	

SX-F-COMT1	*COMT1*	TTGATCTCCCACATGTCATCG
SX-R-COMT1		ATGTTCGTCACTCCAGTCATG
LT-F-CCOAOMT	*CCOAOMT*	CTCAGGGAAGTGACAGCAAA
LT-R-CCOAOMT		GTGGCGAGAAGAGAGTAGCC
LT-F-AHP4	*AHP4*	CTCCAAGATGATGCAAACCCTAA
LT-R-AHP4		TCAGCTTTCACTTTACTTGCCC
SX-F-CAD4	*CAD4*	ATGTCTAATTATCCTATGGTTCCTGG
SX-R-CAD4		ACTCCGACTACATCTCCTACG
SX-F-CAD5	*CAD5*	CATCAATGGTCAACCTACACAAG
SX-R-CAD5		TCAACCGCCATTCCTTCTG
SX-F-CAD6	*CAD6*	TTGGGACGAAAATCGATAGC
SX-R-CAD6		TGCTTTTATGCCATGCTCTG
SX-F-HCT	*HCT*	GCTCTTAAGGCGAAATCCAAG
SX-R-HCT		CTTTCCCACTGATCTCCACAC
SX-F-CCR1	*CCR1*	ACCAAGTGCAAGGACGAGAA
SX-R-CCR1		GTCGTAGAGGCTTTGCTTGG
SX-F-F5H1	*F5H1*	TCCATCAAACTTACCCGTGAC
SX-R-F5H1		TGTTGGACCCGTTTTAGATCC
LT-F-NST1	*NST1*	AAGCATCACAAGACGAAGGATG
LT-R-NST1		ATGATGTCGTCTTCGGCGTAT
LT-F-NST2	*NST2*	CAATGAGGAGACTATCGAGCAA
LT-R-NST2		TGATAACTCGTGATGGTGGTGT
LT-F-SERK2	*SERK2*	TCGTTATGGCTTGCCTCTTC
LT-R-SERK2		CCAGACAAGTCTGCATTCCC
LT-F-BAM1	*BAM1*	TGGCTCTTACGGCTACATAGC
LT-R-BAM1		GTCTCCAAATTCTCCGACAGG
LT-F-BAM2	*BAM2*	CCTACGGCTACATAGCTCCAG
LT-R-BAM2		AATCCGTCATGCTTCTCACCC
LT-F-BIM1	*BIM1*	CAACCATATGAGCGGTTTCAG
LT-R-BIM1		ATGGCTCTGGCTAGTCGAGG
LT-F-MYB33	*MYB33*	CCGAGGAGTTTGACAATTCCC
LT-R-MYB33		TCCCTAAACCATGGTCAAGCC
LT-F-MYB65	*MYB65*	TGAAATGCACGCCAAGATGGG
LT-R-MYB65		GTAAGCCTGCTCGTTGTCTCC
LT-F-TPD1	*TPD1*	GCTCTACGGCATGGCTCTCG
LT-R-TPD1		CTCCGGCTCTACACTCCTTTC
LT-F-SPL11	*SPL11*	CAATGAATCCCGAGAGGGTG
LT-R-SPL11		TTACCTCTCTGGAAGCTCAG
LT-F-SPL8	*SPL8*	CACAGAAGGCACAAAGTG

LT-R-SPL8		GGTTATGGTCAGCAAGTC
LT-F-SPL9	*SPL9*	CTCGAGACACCGAGTTTGTGG
LT-R-SPL9		CGTCGCTCATTATGACCAGCG
LT-F-SPL2	*SPL2*	CGGTTCTGCCAACAATGTAGC
LT-R-SPL2		ATGGTACGTGCTTCGAACTTG
LT-F-SERK1	*SERK1*	GCAGAGCTGGGATCCTACGC
LT-R-SERK1		TTGAGCACACCAAGCTCTGG
LT-F-DYT1	*DYT1*	TGTCGGCTTATGGCTCTGCG
LT-R-DYT1		AATCTCAGGAGGAGCTTCTTCC
LT-F-ROXY1	*ROXY1*	GGTGGCTGACACTGGTCTGC
LT-R-ROXY1		GGTGGAGGTCGAGCTCGTGG
LT-F-ROXY2	*ROXY2*	CATGAATGTGTTTCCGTCGGAG
LT-R-ROXY2		CAACTCCGTAAGGGAGGAGGTC
LT-F-TGA9	*TGA9*	CAAGTATAGGAACGAGGAAGCC
LT-R-TGA9		GATCCTGAATCGCTGCTC
LT-F-TGA10	*TGA10*	GGACAAGGACATCATGATCCTC
LT-R-TGA10		GTGTGTAGAAGAAGGAGGTGGC
LT-F-NZZ	*SPL/NZZ*	CAGATCATCATCAGAGGAGCC
LT-R-NZZ		GTTTCCTTCCATGTAGCTCCC
LT-F-AMS	*AMS*	ATGTTTCATCATCCTCGGACC
LT-R-AMS		CTTGGTTCTGAGCTCTCACTC
LT-F-EMS1	*EMS1*	CCTCAGCTTCTCTACCTCGAC
LT-R-EMS1		ATCTCCGACGGAATTTGACC
18S RNA-F	*18S rRNA*	CGTCCCTGCCCTTTGTACAC
18S RNA-R		CGAACACTTCACCGGATCATT
RAN3-F	*RAN3*	ACCAGCAAACCGTGGATTACCCTAGC
RAN3-R		ATTCCACAAAGTGAAGATTAGCGTCC

Plasmid Construction and Transgenic plants

The *pUBQ10:TCP5* and *pUBQ10:TCP5-SRDX* constructs were created using gateway cloning. The full-length coding sequence (CDS) of *TCP5* was amplified by PCR from wild-type Arabidopsis inflorescence cDNA using primer pair (5′-GGGGACAAGTTTGTACAAAAAAGCAGGCTTAATGAGATCAGGAGAATGTGATGAAG-3′ and 5′-GGGGACCACTTTGTACAAGAAAGCTGGGTGAGAATCTGATTCATTATCGCTAC-3′), and the *TCP5-SRDX* sequence (*TCP5*-CDS-without stop codon fused with CTTGATCTTGATCTTGAACTTAGACTTGGATTTGCTTAA, a sequence encoding EAR-motif with stop codon) was synthesized directly by Sangon Biotech, Shanghai. These sequences were

added to pDONR207 separately using BP reactions (Gateway™ BP Clonase™ II enzyme mix , Invitrogen/Thermo Fisher Scientific, Shanghai, China) and then transferred into the destination vector pUB-Dest [44] using LR reactions (Gateway™ LR Clonase™ II enzyme mix, Invitrogen/Thermo Fisher Scientific, Shanghai, China). For the BP and LR reactions, the standard reaction setup includes: PCR product/synthesized DNA sequence: 100 ng; entry/destination expression vector: 100 ng; BP/LR Clonase II enzyme: 1 μL; ddH_2O to adjust the total reaction volume of 5 μL. This mixture was incubated at 25 °C for 1 h, followed by direct TOP10 competent cells transformation.

A *pOLE:OLE-TagRFP* cassette expressed in seeds from a pDe-CAS9 derivative [73-75] was cut off using SmaI and PstI, and integrated into *pUBQ10:TCP5* and *pUBQ10:TCP5-SRDX*, respectively, to allow for visual screening of the transgenic plants. The 2.1 kb *SPL8* promoter, expressed in early anther [61], was acquired via PCR (primer pair: 5′-GCaggcctTATGCATAATACTTGCGCAG-3′ and 5′-TGTATctcgagTTGCAATCCCGAGGAAGATCTCTCTC-3′, StuI and XhoI targeted sites in lowercases were added near their 5′-ends in the primers, respectively) from wild-type leaf genomic DNA for the synthesis of *pSPL8:TCP5-SRDX*, and the *pUBQ10* promoter in *pUBQ10:TCP5-SRDX* was replaced through appropriate restriction enzyme digestion and ligation. A 5173 bp *TCP5* promoter was amplified (PCR primer pair: 5′-AGtctagaTTAGTATTGCATATAGCCTATAC-3′ and 5′-TGCAcccgggCTCTTTAATTCACTCAACAAG-3′, XbaI and SmaI targeted sites in lowercases were added near their 5′-ends in the primers, respectively) on the wild-type genomic DNA for *pTCP5:GUS* construction, and it was digested using XbaI and SmaI, and then inserted into the pGPTV-BAR [76] just before the β-glucuronidase (*uidA*) reporter gene. PCR, restriction enzyme digestion, and sequencing validated the plasmids.

These plasmids were then transformed into wild-type plants by floral dipping using *Agrobacterium tumefaciens* strain GV3101 [77]. Briefly, 1 μg of plasmid mixed into 100 μL GV3101 competent cells, frozen in liquid nitrogen for 2 min, and followed by a heat shock at 37 °C for 5 min. The mixture was added into 1 mL of liquid YEB medium and incubated at 28 °C in a shaking incubator at 180 rpm for 2 h. The incubated suspension was then spread on a solid YEB plate containing gentamicin (Gent, 25 μg/mL), rifampicin (Rif, 50 μg/mL), and the appropriate antibiotics for the plasmid, growing at 28 °C for 48 h. After preparation of the Agrobacterium solution for transforming the plants, 5-6 weeks old Arabidopsis plants with a few opening flowers were selected for dipping. Basta spraying or screening red seeds with a fluorescence stereomicroscope helped in the selection of transgenic plants (Leica M205C; Leica Microsystems, Mannheim, Germany).
